# Low‐rank iterative infilling for zero echo‐time (ZTE) imaging

**DOI:** 10.1002/mrm.30345

**Published:** 2024-11-04

**Authors:** Zimu Huo, José de Arcos, Florian Wiesinger, Joshua D. Kaggie, Martin J. Graves

**Affiliations:** ^1^ Department of Radiology University of Cambridge, Cambridge Biomedical Campus Cambridge UK; ^2^ GE HealthCare Amersham UK; ^3^ GE HealthCare Munich Germany; ^4^ Department of Neuroimaging, Psychology and Neuroscience King's College London London UK

**Keywords:** dead‐time gap, low rank, ZTE

## Abstract

**Purpose:**

A new referenceless low‐rank reconstruction technique has been introduced to address the issue of missing samples within the Zero Echo Time (ZTE) dead‐time gap.

**Methods:**

The proposed method reformulates the in‐filling of the missing samples as an inverse problem subject to low‐rank constraints. Its performance and robustness are evaluated through a comparative analysis that combines Monte Carlo computational simulations and data obtained from in vivo experiments.

**Results:**

The proposed method is tested for dead‐time gaps ranging up to 4.5 Nyquist dwells, across signal‐to‐noise ratio levels of 5, 10, 15, and 20 dB. Consistently superior performance is observed across all cases compared to algebraic and parallel imaging methods. The speed for convergence decreases exponentially as the dead‐time gap expands.

**Conclusion:**

The proposed method enables artifact‐free reconstruction up to dead‐time gap of 4 Nyquist dwells and thereby supports ZTE imaging up to an imaging bandwidth of ±41.67 kHz (assuming transmit and receive switching less than 30 μs). It demonstrates superior performance compared to algebraic and parallel imaging methods.

## 1 INTRODUCTION

Methods for imaging tissues with short and ultra‐short transverse relaxation times ($T_{2}^{*}$), on the order of a few milliseconds, have gained increasing attention for their potential in clinical diagnosis and research.^1^, ^2^, ^3^, ^4^ In recent years several specialized sequences have been developed to capture signals from tissues characterized by extremely short $T_{2}^{*}$ components including bone, tendon, myelin, lung, and teeth.^5^, ^6^, ^7^, ^8^, ^9^, ^10^, ^11^, ^12^, ^13^, ^14^, ^15^ Unlike conventional MR methods, which lack signal from short $T_{2}^{*}$ tissues, zero or ultrashort echo time sequences allow for the measurement of ultra‐short $T_{2}^{*}$ tissues within a range of 10 μs to 1 ms.^15^, ^16^, ^17^, ^18^, ^19^, ^20^, ^21^

One effective technique is Zero Echo Time imaging (ZTE),^22^ where radiofrequency (RF) excitation is performed during spatial encoding. Maximum scan efficiency is obtained by reducing the time between RF excitation and signal acquisition. These immediate short readouts provide improved sensitivity to short‐short $T_{2}^{*}$ signals while minimizing sensitivity to $B_{0}$ off‐resonance and eddy currents, albeit at the expense of nonselective excitation. However, the application of an encoding gradient during the short RF excitation pulse leads to a dead‐time gap, i.e., missing samples, in the center of *k*‐space. The number of missing samples increases when wider imaging bandwidths are used. These missing Fourier coefficients can result in spatially varying signal modulations due to convolution with a sinc function.

In ZTE imaging, increasing the imaging bandwidth reduces chemical shift artifacts and improves overall image quality.^23^ On our clinical MR system, the shortest achievable dead‐time gap, corresponds to three missing samples along each radial spoke using a readout bandwidth of ±31.25 kHz (or five missing samples on a two times oversampled grid). At a wider bandwidth of ±62.5 kHz, this increases to five missing samples (nine missing samples on a two times oversampled grid). This increase in the dead‐time gap affects image quality and necessitates in‐filling of the missing samples.

Several sequence‐based solutions have been proposed to address the missing points in the free induction decay. The Water‐ and Fat‐Suppressed Proton Projection MRI (WASPI) method uses a separate short acquisition with reduced amplitude gradients, i.e., a lower bandwidth, to sample the missing samples.^24^ The pointwise encoding time reduction with radial acquisition uses a relatively slow single‐point acquisition method.^25^ In an attempt to balance acquisition time and image quality, Hybrid Filling of the Dead‐Time Gap for Faster Zero Echo Time Imaging method merges WASPI and pointwise encoding time reduction with radial acquisition techniques.^26^ All these techniques introduce an nonideal point spread function (PSF) and potentially cause phase inconsistencies due to motion when combined with ZTE data.

Another group of methods based on image reconstruction and data recovery have also been proposed to infill the dead‐time gap, often using the acquired data to synthesize the missing samples. One common approach is the algebraic method,^27^ which utilizes the idea of explicitly filling the missing data points by leveraging information outside the dead‐time gap. This method formulates a system of linear equations based on readout oversampling for estimating the missing points. This has been shown to be computationally efficient as these equations only need to be solved once. However, diametric spokes are needed, which require that each radial center‐out spoke has a mirrored opposite counterpart, reducing the acquisition efficiency, and the reconstruction becomes ill‐conditioned when dead‐time gaps exceed three missing points.^28^ The Stoch–Olejniczak algorithm specifically targets minimizing the real signal outside the nominal field‐of‐view (FOV) instead of minimizing the norm of the signal as for the algebraic method.^29^, ^30^ Recently, parallel imaging‐based algorithms inspired by generalized auto‐calibrating partially parallel acquisition (GRAPPA)^31^, ^32^ and sensitivity encoding technique (SENSE)^33^, ^34^ have been introduced to fill the dead‐time gap. The ZINFANDEL method involves using a *k*‐space interpolation kernel to fill the missing data using the coil sensitivity information, that is, the kernel exploits local *k*‐space correlations across coil channels.^32^ The kernel is trained on a small subset of radial spokes in close proximity to the dead‐time gap. As interpolation is directly performed along each radial spoke, the kernel weights vary with angle, requiring recalibration of these weights for each missing data point. The conjugate gradient SENSE (CG SENSE) method demonstrates the implicit filling of small dead‐time gaps during iterative reconstruction, much like filling missing lines of *k*‐space in traditional parallel imaging.^34^

Here, we propose to reformulate the ZTE dead‐time infilling as an inverse problem subject to low‐rank constraints. The proposed method leverages the finite support information acquired through twice‐readout oversampling used in algebraic reconstruction techniques. It further refines this support using low‐rank techniques by recognizing that the image does not occupy the entire nominal FOV, thereby allowing for a potentially narrower support. This approach also integrates coil sensitivity information, akin to the methods employed in ZINFANDEL and CG SENSE, to inform the reconstruction. We used the P‐LORAKS framework modified for non‐Cartesian data. Additionally, we demonstrate that only low‐resolution data is required for the infilling task, significantly reducing computational burden and memory usage. This study begins with computer simulations aimed at optimizing the reconstruction parameters for the proposed method. Subsequently, we conducted a comparative analysis between our proposed method and established techniques, such as the algebraic method, Stoch–Olejniczak algorithm, ZINFANDEL, and CG SENSE, evaluating their performance across various gap sizes and noise levels. Finally, we validate these methods through in vivo acquisitions.

## 2 THEORY

### 2.1 ZTE pulse sequence and dead‐time gap

**FIGURE 1:**
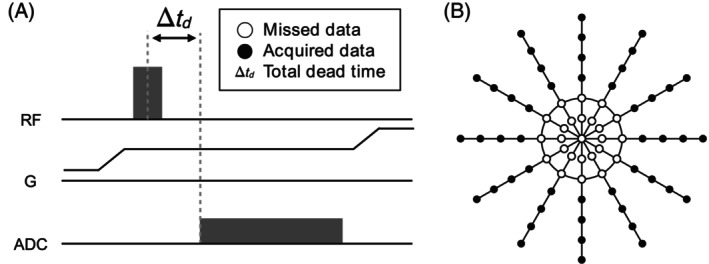
Schematic of a zero echo time (ZTE) sequence for (A) one repetition time and (B) the three‐dimensional radial ZTE trajectory in the x‐y plane. The total dead‐time is determined by the radiofrequency (RF) pulse duration and the RF amplifier blanking time. This gap prevents several low‐frequency Fourier coefficients from being acquired, which in turn can lead to substantial reconstruction artifacts.

The ZTE sequence maintains a constant gradient amplitude throughout a single repetition time (TR).^22^ Once the desired gradient amplitude is reached, a short duration hard RF pulse is played. Following this, the coil switches from transmit to receive mode acquiring the free induction decay signal as shown in Figure 1. However, due to the finite RF pulse duration and the time taken for RF switching, it is not possible to sample the free induction decay near the *k*‐space origin. This limitation imposes a finite dead‐time $\Delta t_{d}$, during which the signal cannot be sampled. The time missed during data acquisition is referred to as the dead‐time, and the number of missing samples is calculated as the ratio of the dead‐time $\Delta t_{d}$ and the dwell time $d_{t}$ = 1/RBW, where RBW is the total receiver bandwidth. In this work, the dead‐time gap is always referred to as the number of missing samples, i.e., Nyquist dwells, on the nominal grid with no oversampling factors.

**FIGURE 2:**
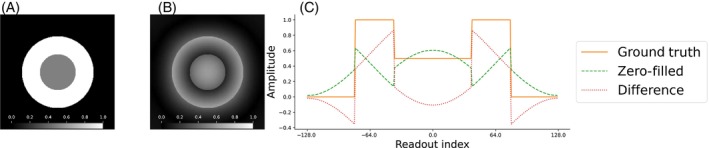
Overview of the effects of the dead‐time gap in the two‐dimensional simulated spherical phantom. The image comprises a white circle (radius: 80 pixels) and a gray circle (radius: 40 pixels), both centered within a 256 by 256 pixel‐sized image. For simplicity, the image is undersampled with a radial mask that nulls the central Fourier coefficients within a radius of three samples. (A) The ground truth magnitude image. (B) The corrupted image, illustrating a dead‐time gap of two samples with no oversampling. (C) The one‐dimensional projection of the ground truth signal from a single spoke pair. (A) Ground truth; (B) zero‐filled; (C) one‐dimensional projection.

Figure 2 demonstrates the effect of a dead‐time gap of two Nyquist dwells on a simulated two‐dimensional (2D) image. In Figure 2C, the one‐dimensional (1D) projections of the ground truth, zero‐filled data, and their difference, derived from a single spoke pair are presented. The net effect of setting the center three Nyquist dwells to zero is equal to convolving the ground truth image with a sinc function, resulting in ringing artifacts around the object. The absence of the low‐frequency Fourier coefficients translates, through Fourier transformation, into amplitude modulations within the image domain. This leads to a considerable reduction in image contrast.

### 2.2 Algebraic method

In the algebraic method, the reconstruction aims to recover the missing samples from each radial spoke and its opposite counterpart.^27^ The 1D projection of the image will be represented by a vector $\hat{x}$. The image is acquired with multiple receiver coils denoted by a sensitivity encoding operator S. The Fourier encoding of each radial spoke is represented as a discrete Fourier encoding matrix F, where each row captures the positive or negative frequency attributes within a signal. The dead‐time gap within the ZTE radial sampling can be denoted by a diagonal sampling mask, referred to as D. In this representation, elements obtained through sampling are denoted as one, while those located within the dead‐time gap are assigned a value of zero. Consequently, the forward model can be succinctly expressed using the above sequence of linear operators:

(1) $$ŷ=\text{DFS}\hat{x}+n.$$

**FIGURE 3:**
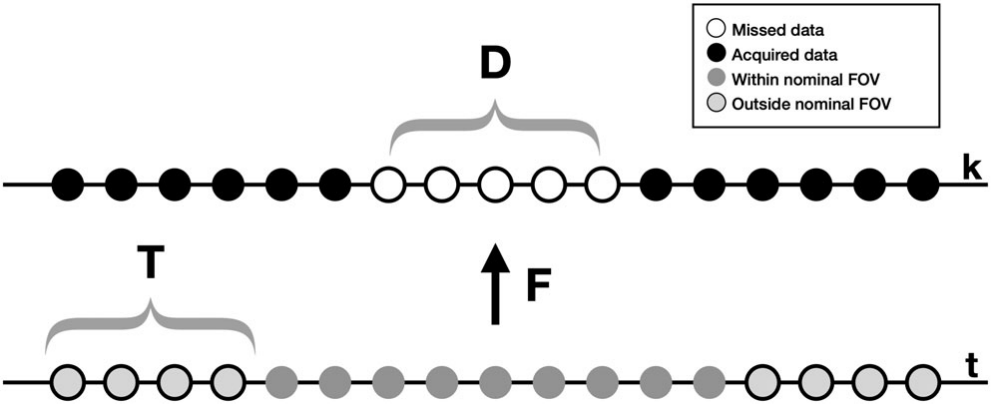
The illustration of the algebraic and Stoch–Olejniczak methods. In this context, the image domain is denoted by “t,” while the *k*‐space domain is denoted by “k.” In the algebraic method, a system of linear equations is established to identify the missing low‐frequency k‐space coefficients (in region D) that minimize the norm of the signal outside the nominal field of view (in region T). The Stoch–Olejniczak method determines the optimal coefficients that minimize only the real part of the signal in region T.

Here, ŷ signifies the 1D radial *k*‐space data and *n* represents the complex white Gaussian noise inherent in the measurements. When data is collected at a frequency higher than the object's bandwidth, the object only occupies a specific area in the FOV, allowing us to assume that outside this region, values should be zero. By using an oversampled grid, we can represent the knowledge that regions beyond the regular frequency band should be zeros using a diagonal matrix T, where one corresponds to elements within the frequency band, and zero corresponds to elements outside the band. A graphical illustration of the ZTE algebraic reconstruction is provided in Figure 3.

(2) $$ŷ=\text{DFTS}\hat{x}+n.$$

The algebraic method relies on solving the inverse of the system matrix A = DFTS. This process is repeated for all radial spokes and their opposite counterparts and reconstructed on a per‐coil basis. To reduce sensitivity to noise, additional regularization techniques such as Tikhonov regularization and truncated singular value decomposition (SVD) can be employed.^27^, ^28^

### 2.3 Stoch–Olejniczak method

The Stoch–Olejniczak algorithm, akin to algebraic methods, employs image support from two times oversampling to estimate missing data.^29^, ^30^ In algebraic reconstruction, a system of linear equations is formulated by identifying the missing low‐frequency *k*‐space coefficients that minimize the norm of the signal outside the nominal FOV. Conversely, the Stoch–Olejniczak algorithm exclusively minimizes the real signal outside the nominal FOV, potentially providing immunity to any phase errors. Another notable distinction is its requirement for only a single spoke, rather than a radial spoke pair.

### 2.4 ZINFANDEL

Recently, the ZINFANDEL method has been introduced, drawing inspiration from the GRAPPA parallel imaging algorithm to address missing samples.^31^, ^32^ In the original Cartesian GRAPPA reconstruction method, the challenge of reconstruction is formulated as an interpolation problem in *k*‐space, where neighboring data points are utilized to fill the missing samples in *k*‐space. GRAPPA involves training a *k*‐space interpolation kernel using fully sampled low‐resolution calibration data. The calibration process seeks a set of weights that best aligns with the calibration data through a least‐squares approach. ZINFANDEL, however, operates differently by directly applying the interpolation kernel to the radial spokes. The calibration data is obtained directly from sampled radial data points in proximity to the dead‐time gap. The calibration region is kept small to ensure the kernel's angular variation remains minimal, maintaining stability in its operation on the radial data points.

### 2.5 CG SENSE

The dead‐time gap can also be filled implicitly during iterative CG SENSE reconstruction reformulated as follows^34^:

(3) $$\underset{x}{\min}\;║\text{DFS}x-y{║}^{2}+\lambda ║x{║}_{2}.$$

The vector *x* is obtained by flattening the true image. In this case the operator *F* denotes the nonuniform Fourier transform. This can be solved using normal equation, but the inverse of the system matrix DFS is computationally demanding, the problem is typically solved in an iterative fashion. A numerically fast method to solve problem subject to the L2 norm is given by the conjugate gradient algorithm (Algorithm 1).

### 2.6 Low‐rank iterative infilling

Algorithm 1 **Low Rank Iterative Infilling for ZTE Data**

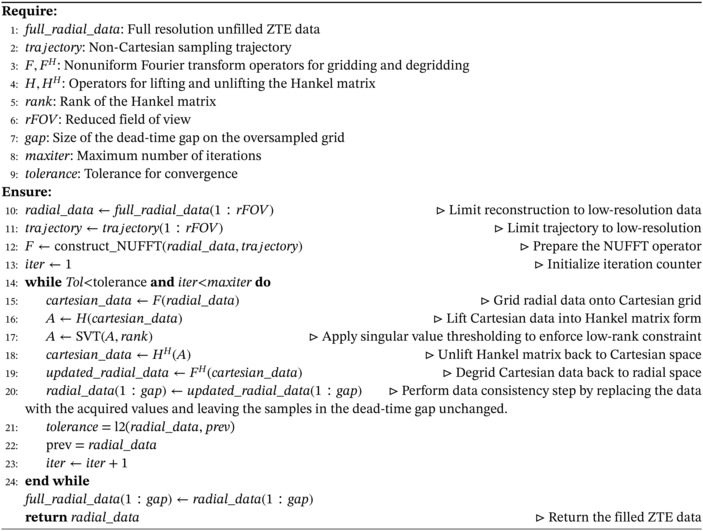

The theory of structured low‐rank interpolation and its relationship with an annihilating filter has been extensively studied.^35^ If the image x has finite support within the FOV, then there exists a smooth spatial domain annihilating function ĥ, which is nonzero outside the support of the image x and zero within the finite support region. Consequently, when this filter is applied to the image, the product is zero, hence,

(4) x·ĥ=0.

Using the duality of Fourier transform, the spatial domain multiplication translates into a convolution with h in the Fourier domain,

(5) (F·x)∗h=0.

The ∗ symbol denotes convolution. Since ĥ is spatially smooth by definition, the filter h is band‐limited. Equations of this form, where one side is set to zero, are referred to as annihilation relations. The data x can be predicted using a local linear model from the shift invariant coefficients of the filter h (i.e., predict the value of the target coefficient using the weighted sum of other samples in the local neighborhood of k‐space). For instance, consider a local *k*‐space region consisting of three samples, denoted as $k_{1}$, $k_{2}$, and $k_{3}$, with corresponding filter coefficients $w_{1}$, $w_{2}$, and $w_{3}$, such that $\sum_{i=1}^{3}w_{i}k_{i}=0$ (i.e., annihilation). Given all the filter coefficients and knowing the values of samples $k_{1}$ and $k_{2}$ we can explicitly determine the missing sample $k_{3}$ as follows: $k_{3}=-\frac{w_{1}k_{1}+w_{2}k_{2}}{w_{3}}$. Structured low‐rank algorithms aim to implicitly determine the local relationships in *k*‐space by using a low‐rank Hankel matrix of the data as a prior (without explicitly computing the filter coefficients). To do this, the convolution in Equation (5) can be rewritten as matrix multiplication, which requires each small *k*‐space patch at each *k*‐space location to be flattened into a row vector and concatenating all the row vectors to form a 2D Hankel matrix. This process is referred to as the Hankel transformation, which we will denote by the symbol H,

(6) (HFx)×h=0.

The Hankel matrix exhibits a null‐space vector associated with the filter coefficients h. Given that it is possible to derive a large set of linearly independent equations as seen in Equations (5) and (6), the Hankel matrix consequently possesses a nontrivial null‐space and is low rank.

The low‐rank‐based method will recover the missing data by enforcing self‐consistency among neighboring *k*‐space points in Cartesian space when minimizing the rank of the structured Hankel matrix. The self‐consistency refers to the annihilation relationship being satisfied for all locations in *k*‐space. Directly solving rank problems is computationally challenging and falls under the category of non‐deterministic polynomial time (NP)‐hard problems.^36^, ^37^ To address this, the nonconvex rank function is replaced with its convex relaxation, which is replacing the nonconvex rank function with its convex approximation, known as the nuclear norm.^38^ Thus, the problem of the missing data in the center of *k*‐space is reformulated as a structured locally low‐rank nuclear norm minimization problem, as expressed by the following:

(7) $$\underset{x}{\min}\;║\text{DFS}x-y|{|}^{2}+\lambda ║\text{HFS}x|{|}_{*}.$$

**FIGURE 4:**
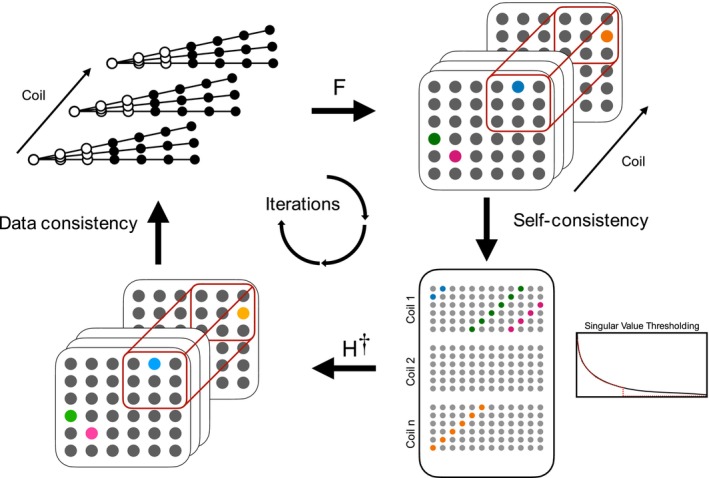
Schematic of the proposed low rank reconstruction. The z‐dimension is ignored in the flow chart for simplicity. To enforce self‐consistency, the Cartesian data is reorganized into a structured Hankel matrix, where singular value thresholding is performed to shrink the singular values to a predefined range. Data consistency is enforced by transforming the Cartesian data back to the non‐Cartesian space, where synthesized data is replaced with the acquired measurements.

Here, *H* represents the matrix lifting operator, which converts three‐dimensional (3D) Cartesian data into a structured Hankel matrix and * denotes the nuclear norm. The P‐LORAKS framework necessitates that data be structured on a Cartesian grid. Therefore, we start by converting the non‐Cartesian radial data into a 3D Cartesian grid. Subsequently, the Cartesian data are organized into GRAPPA‐like kernels. Each kernel is then flattened, creating columns within a 2D Hankel matrix, as described in prior studies.^38^, ^39^ This process is iterated by convolving the kernel across either the entire *k*‐space or a predefined center region. In instances where a fully sampled ZTE acquisition is in use, only the center data is absent, thus only the center volume is needed. To enforce self‐consistency and low rankness, we apply the Singular Value Thresholding to the Hankel matrix, employing either hard or soft thresholding on the singular values. Subsequently, the Cartesian data is inverse‐transformed into non‐Cartesian space to maintain data consistency by replacing the data with the acquired data. The operation as indicated by Figure 4 is iteratively repeated until convergence is achieved. This can be solved efficiently using Projection‐onto‐Convex‐Sets algorithm.^38^, ^39^ The pseudo‐code is provided in Algorithm 1.

## 3 METHODS

### 3.1 3D simulation

To compare the performance of the infilling methods, we conducted computer simulations employing a 128 × 128 × 128 digital brain phantom.^40^ The 3D brain dataset was then encoded with simulated 3D four‐channel receive coil profiles. The code used to simulate the coil profiles are obtained from Michigan Image Reconstruction Toolbox (MIRT).^41^ This Cartesian dataset was subsequently sampled utilizing a 3D radial phyllotaxis trajectory, modified to sample the opposing radial spoke.^42^

Given the computational intensity inherent in full 3D datasets, reducing the computation cost of a nonuniform Fourier transform (NUFFT) and SVD becomes imperative. We first perform parameter optimization for the proposed low‐rank reconstruction, specifically assessing the reconstruction performance as a function of the center volumetric dimension, given that the dead‐time gap impacts solely the central portion of *k*‐space. This optimization is done by conducting an exhaustive search across potential volume dimensions, ranging from 5 to 64, to observe the associated reconstruction performance.

Following the parameter optimization, this study then commences by investigating the infilling performance in relation to different gap sizes, while also considering its performance dependence on the system noise levels. The radial data was radially undersampled with varying dead‐time gap sizes from 1 to 4.5 Nyquist dwells. Additionally, complex white Gaussian noise to simulate signal‐to‐noise ratios (SNRs) of 5, 10, 15, and 20 dB was introduced to the radial *k*‐space data to evaluate the robustness of the reconstruction methods. This noise was added along the radial spoke. SNR calculations are based on the ratio, in logarithmic scale, between the average squared magnitude of the noise‐free image and that exclusively stemming from the noise in the realization:

(8) $$\text{SNR (dB)}=10\log_{10}\left(\frac{\sum x^{2}}{\sum {\left(F^{H}𝒩(\mu ,\sigma^{2})\right)}^{2}}\right),$$

where μ is the mean of the generated complex white Gaussian noise and σ is the noise SD. The mean is set to zero, and the σ is determined through manual tuning based on the desired SNR levels. Identical noise is applied across all correction methods within a single realization. Each reconstruction technique is assessed using 20 different noise realizations for each SNR level and gap size, and the averaged result is reported.

### 3.2 In vivo imaging experiments

The method was evaluated using acquisitions from a healthy volunteer, with UK ethical approval.Informed consent was obtained from the volunteer. The data was acquired on a 3.0 T scanner (Signa Premier XT, GE HealthCare, Waukesha, WI) using a 48‐channel head‐coil. The in vivo brain image is acquired with an isotropic spatial resolution of 0.89 mm and a FOV of 235 mm. The product ZTE acquisition, using WASPI for the center of *k*‐space, served as the ground truth for the in vivo acquisition. The bi‐directional sampling paths have been adapted from Archimedean spirals to sample the opposing radial spokes. The flip angle was set to 1°, and the readout bandwidth was set to ±15.6, ±20.8, ±31.25, ±41.67, and ±55.56 kHz with two times readout oversampling. Dead‐time gaps of 2, 2.5, 3, 3.5, and 4.5 Nyquist dwells are introduced under this setting, respectively. Coil compression is used to reduce the coils from 48 channels down to eight channels.^43^

### 3.3 Image reconstruction

The reconstruction was conducted offline using Matlab (MATLAB and Statistics Toolbox Release 2022b, The MathWorks, Inc., Natick, MA). The non‐Cartesian data was reconstructed on a 2× oversampled grid. We used in‐house implementations of algebraic, ZINFANDEL, and low‐rank methods. The Stoch–Olejniczak method is obtained from a Github open‐source implementation.^30^ For the ZINFANDEL reconstruction, we employed kernel parameters as proposed by the original author, utilizing a calibration size comprising of 16 readout samples within the nearest five radial spokes, alongside a 1D kernel length of 5.^32^ The CG SENSE reconstruction is modified based on the gpuNUFFT Matlab package.^44^ For the algebraic, Stoch–Olejniczak, ZINFANDEL, and CG SENSE, we applied Tikhonov regularization. The optimal value of Tikhonov regularization was found using a parameter sweep between λ∈[0,1e−10].

We implemented the P‐LORAKS C‐matrix with a 3D isotropic kernel of size 5 as the matrix lifting operator.^35^, ^39^, ^45^ A hard threshold singular value threshold was employed that zeroed out singular values below 1.5% of the highest singular value. Only the first 20% of each radial spoke were used to grid the data onto a low resolution *k*‐space Cartesian grid of 26 × 26 × 26. These parameters have been observed to perform well for gap sizes less than 4.5. For the NUFFT operation, we used a conjugate gradient based NUFFT operation with 10 iterations for all reconstruction methods. All reconstructions differ only by the Fourier coefficients within the dead‐time gap; the region outside the dead‐time gap was kept constant.

The reconstruction error is calculated using the squared error of the ground truth magnitude image a and the reconstructed magnitude image b:

(9) $$\|a-b{\|}_{2}=\sqrt{\overset{n}{\underset{i=1}{\sum }}{\left(a_{i}-b_{i}\right)}^{2}}.$$

## 4 RESULTS

**FIGURE 5:**
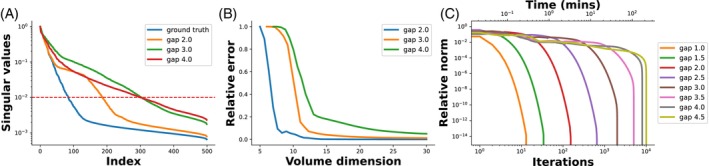
Effect of dead‐time gap on low‐rank reconstruction. (A) The singular values at every even dead‐time gaps while omitting the visualization of the odd ones for clarity. (B) The relationship between the performance of low‐rank reconstruction and the central volume dimension. Notably, a small portion of the central volume suffices for artifact‐free reconstruction, ensuring optimal efficiency. (C) The convergence rate in relation to different dead‐time gaps. Convergence is assessed by quantifying the energy difference between the current estimation and the ground truth in k‐space, measured through the sum of the L2 norm of the real and imaginary components.

Figure 5 demonstrates the parameter selection and convergence rate of the low rank infilling method. Figure 5B, demonstrates that only approximately 20%–25% of the data on each radial spoke is required for reconstruction, which greatly alleviates the computational load. Meanwhile, Figure 5C showcases the convergence rate of the low rank method across different gap sizes. The algorithm stops when the relative change in *k*‐space, assessed via Euclidean distance, drops below 1e−6. The necessary steps for the low rank method to converge increases exponentially with each Nyquist dwell.

**FIGURE 6:**
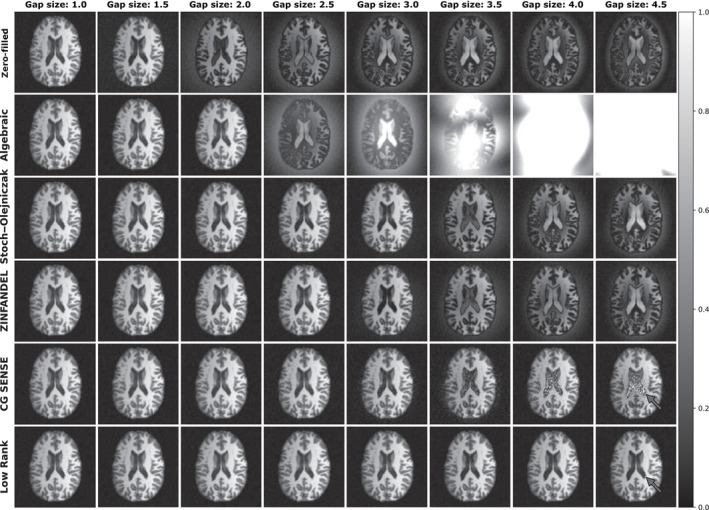
An example of the digital brain simulations across dead‐time gaps ranging from 1 to 4.5 Nyquist dwells at signal‐to‐noise ratio (SNR) of 10 dB. The figure presents the reconstruction outcomes for Zero Filled, Algebraic, ZINFANDEL, and low‐rank methods across dead‐time gaps ranging from 1 to 4.5 Nyquist dwells at an SNR of 10 dB.

Figure 6 shows the low‐rank reconstruction of the simulated digital brain phantom for Zero Filled, Algebraic, Stoch–Olejniczak, ZINFANDEL, CG SENSE, and low‐rank methods across dead‐time gaps ranging from 1 to 4.5 Nyquist dwells at a SNR of 10 dB. The algebraic method starts to fail for dead‐time gaps larger than three Nyquist dwells when it begins over estimating the energy within the dead‐time gap. The CG SENSE and low‐rank methods outperform the algebraic, Stoch–Olejniczak, ZINFANDEL approach, demonstrating superior image quality when infilling larger dead‐time gaps.

**FIGURE 7:**
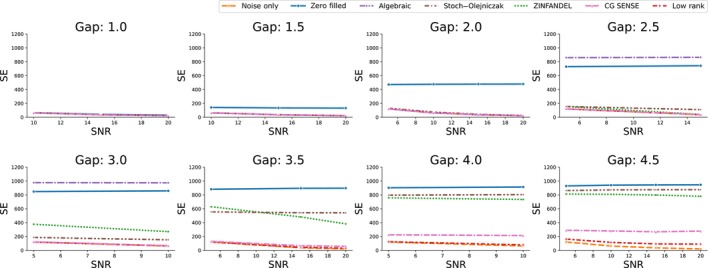
Quantitative analysis of the three‐dimensional brain simulation. The squared errors is plotted against dead‐time gaps spanning 1–4.5 Nyquist dwells at four different signal‐to‐noise ratios (SNRs). Notably, when dead‐time gap exceeds three Nyquist dwells, the squared errors for algebraic method are beyond acceptable thresholds. Consequently, these values are deliberately omitted from the figure to ensure the clarity of the depicted data trends.

Figure 7 illustrates the quantitative analysis of the simulation. When the dead‐time gap remains under two Nyquist dwells, all correction methods effectively fill the missing samples. However, if the dead‐time gap surpasses 2.5 Nyquist dwells, the algebraic method becomes ill‐conditioned, causing the squared error to diverge. The proposed method exhibits artifact‐free reconstruction up to a dead‐time gap up to four Nyquist dwells.

**FIGURE 8:**
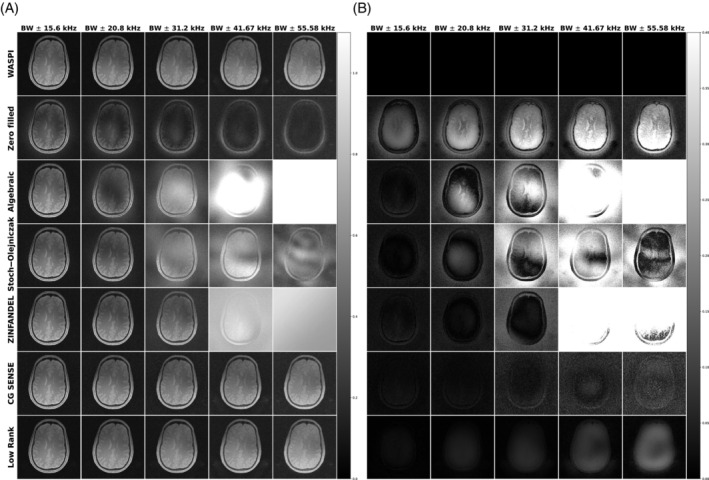
Reconstruction result with data collected using bi‐directional radial sampling path for a normal volunteer. Both the low rank and conjugated sensitivity encoding technique (CG SENSE) methods are effective within a bandwidth of ±41.67 kHz. The CG SENSE method displayed structured noise at bandwidth of ±55.58 kHz. The SNR has been determined to be 15.57 dB for bandwidths of ±15.6 kHz, 14.44 dB for ±20.8 kHz, 13.03 dB for ±31.25 kHz, 11.73 dB for ±41.67 kHz, and 10.02 dB for ±55.56 kHz. (A) Reconstruction; (B) Difference map.

Figure 8 depicts the reconstruction outcomes for in vivo brain data. The image SNR is measured using the signal from the center patch of the brain and the noise from background from the WASPI filled ZTE data. The SNR is estimated to be 15.57, 14.44, 13.03, 11.73, 10.02 dB for bandwidth of ±15.6, ±20.8, ±31.25, ±41.67, and ±55.56 kHz, respectively. The WASPI acquisition serves as a reference to the “elusive” ground truth, due to the challenge posed by a nonideal PSF in all current methods. Notably, CG SENSE and the low‐rank method demonstrate comparable and superior results compared to the algebraic, Stoch–Olejniczak, and ZINFANDEL method. Low‐rank methods offer an additional advantage for filling large gap sizes because they do not introduce extra noise.

## 5 DISCUSSION

The inverse problem presented in this work differs from the conventional parallel imaging reconstruction problem.^31^ Conventional methods involve acquiring a fully sampled low‐resolution region in the center of *k*‐space. This central region is then utilized to estimate either the GRAPPA kernels or coil sensitivity profiles. However, within the ZTE acquisition, this low‐resolution signal is inherently not present. Yet, we have shown that this problem can be solved with low rank, similar to methods used within conventional parallel imaging.

In ZTE imaging, a significant challenge emerges from the large and continuous dead‐time gaps. Unlike conventional parallel imaging techniques that strategically undersample by excluding alternate lines in Cartesian imaging or radial spokes in Non‐Cartesian radial imaging, ZTE has a continuous absence of samples within the dead‐time gaps in the low frequency portions of *k*‐space. Such dead‐time gaps pose challenges as they severely compromise the conditioning of the inverse problem. Furthermore, as the gap size grows, the correlation between the reconstructed data and the originally acquired data diminishes rapidly, introducing significant noise amplification and artifact in the image domain. This ill‐conditioning becomes particularly evident in algebraic methods, where dead‐time gaps exceeding three Nyquist dwells result in complete failure. However, techniques like Stoch–Olejniczak, ZINFANDEL, CG SENSE, and low‐rank methods demonstrate effectiveness in mitigating these challenges. ZINFANDEL and CG SENSE utilize coil sensitivity information within a radial interpolation kernel to efficiently fill larger dead‐time gaps with high accuracy, leveraging more available information. In contrast, the low‐rank method proposed in this work employs both coil sensitivity information and finite image support prior. To address the issue of radial undersampling and further improve the reconstruction, it is possible to introduce additional information, such as virtual coil augmentation, into the low rank reconstruction framework.

In conventional parallel imaging, the acceleration is limited to ×3 per encoding dimension. Beyond ×3 acceleration, noise amplification starts growing exponentially, and can only be compensated via averaging to a certain extent.^46^ In ZTE, the natural oversampling occurring at the center of the radial *k*‐space results in an averaging effect, where the interpolation errors are averaged during the gridding process. So, one would anticipate the ability to bridge larger gap sizes than what is typically assumed by conventional wisdom.

This study categorizes reconstruction methods into implicit and explicit types based on their approaches to infilling the dead‐time gaps. Specifically, the algebraic, Stoch–Olejniczak, ZINFANDEL and low‐rank methods adopt an explicit strategy, confining their infilling solely to dead‐time gaps and thereby preserving the integrity of the sampled signal. In contrast, the CG SENSE method employs an implicit approach, iteratively filling these gaps within the image domain. A notable distinction lies in the artifact distribution: the ZINFANDEL and low‐rank methods induce low‐frequency modulations, which are less perceptible due to their restriction to lower frequencies. In comparison, the CG SENSE method generates artifacts and noise across a broader frequency range, resulting in more conspicuous artifacts in the final output. A summary is presented in Table 1.

**TABLE 1:** Comparison of different reconstruction methods.

Method	Type	Time per reconstruction	Artifacts/noise distribution	Key characteristics
Algebraic	Explicit	∼10 s	Low‐frequency modulation may diverge	System matrix solving once Matrix multiplication per radial spoke pair
Stoch–Olejniczak	Explicit	∼2 min	Low‐frequency modulation	System matrix solving once Matrix multiplication per radial spoke
ZINFANDEL	Explicit	∼1.2 min per Nyquist dwell	Low‐frequency modulation	Training new kernel for each spoke and gap size
CG SENSE	Implicit	∼4 min <5 iterations for gap size <3 More iterations needed for larger gap	Broad frequency structured low‐frequency noise	Requires coil sensitivity profiles
Low‐rank	Explicit	Few seconds per iteration; convergence slows exponentially with larger gaps	Low‐frequency modulation	Only uses the center volume

For both phantom and in vivo experiments, obtaining a genuine ground truth image in ZTE imaging poses challenges due to the dead‐time gap and always on gradient, causing an inevitable deviation of the imaging PSF from the truth. Notably, some residual low spatial frequency differences exist between the reconstruction methods and the reference for the in vivo brain data as shown in Figure 8. This can be attributed, partially, to the different $T_{2}^{*}$ weighting, slice profile, and gradient spoiling effect introduced by the WASPI acquisition.

The iterative reconstruction approach can implicitly fill missing data even without specific constraints at very small dead‐time gaps of one and two. This occurs due to the finite width of the gridding kernel used in the NUFFT operations. Although the absence of low‐frequency Fourier coefficients reduces the total energy of the signal, the missing data is always partially filled when regridded in *k*‐space. Regridding inevitably causes an increase in the overall energy of the image and *k*‐space as the signal within the dead‐time gap is partially filled and samples outside the dead‐time gap are not affected. As a result, when the gap sizes are significantly large, the overall image energy is closer to zero, resulting in a drastically slower rate of convergence. To address this issue, one could integrate a DC estimate using pointwise encoding time reduction with radial acquisition.

The algebraic reconstruction has the quickest computation time as the system matrix requires solving only once. In our CPU‐based implementation, the reconstruction averaged around 10 s. Longer computational time, around 2 min, is observed for the Stoch–Olejniczak method. This is partially due to the need to perform matrix multiplication on every radial spoke, rather than on pairs of radial spokes as is done in the algebraic method. Both algebraic and Stoch–Olejniczak support parallel computing to further improve the speed. The computation time for algebraic and Stoch‐Olejniczak methods is directly proportional to the number and length of radial spokes and is not affected by the size of the dead‐time gap. Our implementation of the ZINFANDEL technique, which required training a new kernel for each radial spoke and gap size, resulted in computation time approximately 1.2 min per Nyquist dwell and scales linearly with the increase in the dead‐time gap. The adaptability of the ZINFANDEL technique extends to filling data gaps, particularly beneficial in cases involving angular under‐sampling. The CG SENSE method requires less than five iterations to converge at gap size smaller than three Nyquist dwells. However, increasingly more iterations are required at large dead‐time gaps. While the computational demands of 3D low‐rank processing can be substantial, focusing solely on the center volume for infilling effectively reduces these demands. Using only the low‐resolution *k*‐space data significantly accelerates NUFFT processing and subsequent SVDs. This modification leads to a considerable reduction in computation time, down to 1.33 s with GPU‐based NUFFT operations per iteration. However, the number of iterations for convergence grow exponentially as the size of the dead‐time gap increases: 0.5 min for a gap of two Nyquist dwells, 1.7 min for a gap of 2.5 Nyquist dwells, and 5.6 min for a gap of three Nyquist dwells.

**FIGURE 9:**
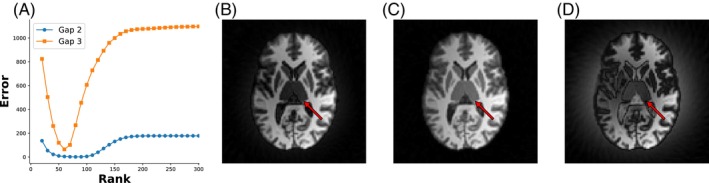
Reconstruction error versus rank. (A) The relationship between reconstruction error and rank. The algorithm demonstrates increased sensitivity to rank as the dead‐time gap increases. (B) Reconstruction outcome when the rank is below the optimal value. (C) The result at the optimal rank. (D) The reconstruction result when the rank exceeds the optimal value. (A) Reconstruction error versus rank; (B) Under estimate rank: 30; (C) Optimal estimate rank: 60; (D) Over estimate rank: 300.

One limitation of the proposed method is its slow convergence rate. The slow convergence rate is attributed, at least partially, to the fact that the low‐rank technique addresses the more challenging task of estimating linear dependence relationships from the Hankel matrix. In contrast, the SENSE‐based technique simplifies this process by explicitly deriving the linear dependence from the prescan data.^47^ To accelerate convergence, one possible approach is to initialize the starting point with other infilling methods. Furthermore, determining the optimal rank necessitates a parameter sweep to identify the optimal rank, which can be time‐consuming. An example of the reconstruction against rank is shown in Figure 9. As the dead‐time gap increases, the algorithm becomes increasingly sensitive to the rank.

We also simplify the scope of this work to fully sampled data. Nevertheless, we anticipate that the proposed method will perform comparably well in undersampled scenarios, as it relies primarily on the low‐resolution center of *k*‐space, where the Nyquist rate is significantly lower. Additionally, this study does not account for factors such as motion artifacts and varying contrast due to different magnetization preparations (e.g., $T_{1}$, $T_{2}$, diffusion). Further investigation is also needed on how low‐frequency modulations affect parametric mapping.^48^ Addressing these factors is beyond the current scope and represents an important aspect for future research.

There will be applications, such as those involving the lung, that require higher bandwidth, where difficulties will remain for any reconstruction process due to limitations in infilling the increased empty data. The use of CG SENSE leads to significant noise amplifications, and the low‐rank approach struggles to restore certain low‐frequency components in the Fourier spectrum.

## 6 CONCLUSION

In our study, we introduce a low rank iterative infilling method for ZTE image reconstruction. This iterative infilling approach enables artifact‐free reconstruction, without the need for gathering additional data and performs well within dead‐time gap 4.5 Nyquist dwells. It demonstrated superior performance than the algebraic, Stoch–Olejniczak, ZINFANDEL, and the CG SENSE method.

## FUNDING INFORMATION

This work was supported by the Gates Cambridge Foundation and the NIHR Cambridge Biomedical Research Centre (NIHR203312*).

## CONFLICT OF INTEREST STATEMENT

Dr. José de Arcos and Dr. Florian Wiesinger are employees of General Electric (GE).

## Data Availability

The reconstruction code for the algebraic, ZINFANDEL, and low‐rank methods, along with example data, can be found in https://github.com/ZimuHuo/LRII.
